# Gut microbiome dysbiosis as a trigger for area postrema syndrome exacerbation in AQP4+ NMOSD with HBV co-exposure

**DOI:** 10.1097/MS9.0000000000004906

**Published:** 2026-04-06

**Authors:** Tahir Ullah, Aneeq Mahmood Khan, Ayesha Mustafa, Mahnoor Fatima

**Affiliations:** a Department of Medicine, Saidu medical college, Swat, Pakistan; b Department of Medicine, Sargodha Medical College, Sargodha, Pakistan; c Department of Medicine, Fatima Jinnah medical university, Lahore, Pakistan; d Department of Medicine, Shaheed Ziaur Rahman Medical College, Rajshahi Medical University, Bogura, Bangladesh

*To the Editor,*

Neuromyelitis optica spectrum disorder (NMOSD) is an autoimmune inflammatory disease of the central nervous system with a presenting sign and symptoms of optic neuritis, longitudinal extensive transverse myelitis, and area postrema syndrome (APS). A recent study in Taiwan with 105 patients with NMOSD shows increased prevalence (51.4%) of past hepatitis B virus (HBV) infection with acquired immunity against HBV in patients with NMOSD, indicating that preceding HBV infection is a risk factor specific to NMOSD. A significantly higher proportion (8.7%) of subjects with past HBV infection was noted in NMOSD, even after following the implementation of the universal vaccination program. Additionally, 5% of the patients with NMOSD in Taiwan (3.8%) and China (3.6%) have chronic HBV infection, while 11.4% of the Taiwanese patients with NMOSD have latent HBV infection. Moreover, past HBV infection was significantly associated with older age at onset and higher disability severity in patients with NMOSD[1]. However, the fact of how past HBV exposure increases disease risk or worsens outcomes remains unclear.

One possible explanation is the gut microbiome. Among them, *Clostridium perfringens* and *Streptococcus* have been confirmed to play a major role in the pathogenicity of NMOSD. There is growing evidence that gut microbiome dysbiosis could be a crucial missing link between AQP4-mediated autoimmunity and prior HBV exposure. A research study from Asian NMOSD populations has revealed that patients’ gut microbiomes are different from those of healthy people, with more pro-inflammatory bacteria and less bacterial diversity[2]. According to the review, AQP4 antibody production and autoimmune inflammation in NMOSD have already been linked to immune pathways like Th17 responses and molecular mimicry, which may be stimulated by aberrant gut bacteria[3]. The area postrema, a part of the brain with high levels of AQP4 and a weak blood–brain barrier, may be particularly affected by this gut–immune interaction. This region may be especially vulnerable to inflammation brought on by an imbalance in the gut microbiome, because it is more exposed to circulating immune signals. This may help explain why immune disorders, including those brought on by previous HBV exposure, can exacerbate symptoms of APS, such as persistent nausea and vomiting[3].

Scientists have identified gut microbiome modulation as a potential treatment method for NMOSD. New research demonstrates that restoring gut microbial balance through various methods, which include probiotics and dietary changes and antibiotics and fecal microbiota transplantation, and other microbiota-based treatments, will help decrease immune system problems and lessen NMOSD symptoms. Probiotics provide body-wide immunomodulation because they help maintain gut wall protection while they boost antimicrobial peptide creation, and they control how the body responds to disease-causing agents. Probiotic strains such as *Streptococcus thermophilus* and *Lactobacillus acidophilus* maintain or increase the phosphorylation of cytoskeletal and tight junction proteins, which results in stronger tight junctions that protect against intestinal mucosal permeability. This epithelial barrier enhancement stops bacteria from entering the body, which prevents subsequent immune system responses. The combined mechanisms demonstrate that probiotics have potential as additional treatment options for immune-mediated disorders that include NMOSD[4]. The gut–brain–immune (GBI) axis comprises a complex communication between the gastrointestinal tract (GI), nervous system, and immune system. Probiotics’ capacity to control immune responses via the GBI axis and show a promising protection and thereby restore health is further supported by data from more extensive neurological studies[5].

The existing evidence shows that people who have had HBV infections before their NMOSD diagnosis will experience disease progression through both immune system pathways and changes to their gut microbiome. The dysbiotic alterations in the gut microbiome will create conditions that boost AQP4-related inflammatory processes, which will mainly affect the area postrema. The investigation of gut microbiome changes in Asian populations who have NMOSD requires long term research studies to establish whether microbiome-based treatment methods will decrease relapse rates in regions where HBV infection remains common. This letter is in line with the TITAN Guidelines on the need for transparency in artificial intelligence use in healthcare[6].

## Data Availability

Not applicable to this study.
